# Supplementary material for: Human forebrain organoid-based multi-omics analyses of PCCB as a schizophrenia associated gene linked to GABAergic pathways
Source: Nat Commun. 2023 Aug 24;14:5176. doi: 10.1038/s41467-023-40861-2 (PMC10449845; doi:10.1038/s41467-023-40861-2)
Supplement: Supplementary file 1 — Supplementary Information [file 41467_2023_40861_MOESM1_ESM.pdf]

Supplementary materials for

Human forebrain organoid-based multi-omics analyses of *PCCB* as a schizophrenia associated gene linked to GABAergic pathways

Wendiao Zhang[1,2,3,4], Ming Zhang[1], Zhenhong Xu[2,3,4], Hongye Yan[2,3,4], Huimin Wang[2,3,4], Jiamei Jiang[2,3,4], Juan Wan[2,3,4], Beisha Tang[2,3,4,6,7], Chunyu Liu[1,5]*, Chao Chen[1,7,8,9]*, Qingtuan Meng[2,3,4,10]*

[1]Center for Medical Genetics & Hunan Key Laboratory of Medical Genetics, School of Life Sciences, and Department of Psychiatry, The Second Xiangya Hospital, Central South University, 410008 Changsha, Hunan, China

[2]The First Affiliated Hospital, Multi-Omics Research Center for Brain Disorders, Hengyang Medical School, University of South China, 421000 Hengyang, Hunan, China

[3]The First Affiliated Hospital, Clinical Research Center for Immune-Related Encephalopathy of Hunan Province, Hengyang Medical School, University of South China, 421000 Hengyang, Hunan, China

[4]The First Affiliated Hospital, Department of Neurology, Hengyang Medical School, University of South China, 421000 Hengyang, Hunan, China

[5]Department of Psychiatry, SUNY Upstate Medical University, Syracuse, NY 13210, USA

[6]Department of Neurology, Xiangya Hospital, Central South University, 410008 Changsha, Hunan, China

[7]National Clinical Research Center for Geriatric Disorders, Xiangya Hospital, Central South University, 410008 Changsha, Hunan, China

[8]Hunan Key Laboratory of Animal Models for Human Diseases, Central South University, Changsha, Hunan 410008, China

[9]Hunan Key Laboratory of Molecular Precision Medicine, Central South University, Changsha, Hunan 410008, China

[10]MOE Key Lab of Rare Pediatric Diseases & School of Life Sciences, University of South China, 421001 Hengyang, Hunan, China

*Corresponding authors:

liuch@upstate.edu (Chunyu Liu); chenchao@sklmg.edu.cn (Chao Chen); mengqingtuan@glmc.edu.cn (Qingtuan Meng)

This supplementary file includes:

Supplementary Fig. S1. Scheme of luciferase reporter vector construction.

Supplementary Fig. S2. Effects of *PCCB* knockdown by *PCCB*-G2 gRNA in U2F hFOs.

Supplementary Fig. S3. Enrichment of 1079 *PCCB* knockdown-induced DEGs with SCZ-related genes.

Supplementary Fig. S4. *PCCB* knockdown leads to mitochondrial dysfunction and decreased GABA levels in U1M hFOs.

Supplementary Fig. S5. MEA assay after *PCCB* knockdown in U1M hFOs.

Supplementary Fig. S6. Expression of GABA receptor genes in ACS-1011 and U1M hFOs.

Supplementary Fig. S7. Immunostaining assay confirmed the existence of GABAergic neurons in hFOs.

Supplementary Fig. S8. Effects of propanoic acid exposure on expression of GABA receptor genes in U1M and U2F hFOs.

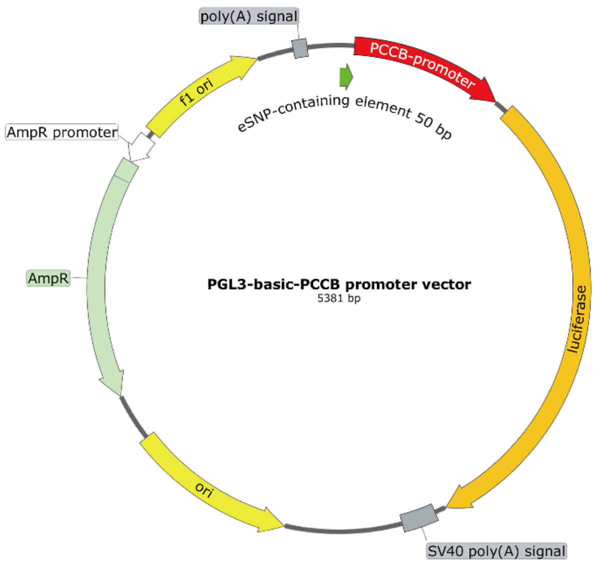

Supplementary Fig. S1. Scheme of luciferase reporter vector construction.

About 50 bp DNA sequence flanking the *PCCB* eSNP was synthesized and cloned into the pGL3-basic luciferase reporter vector. The *PCCB* promoter sequence (~600 bp) was cloned into downstream of the eSNP-containing DNA fragment.

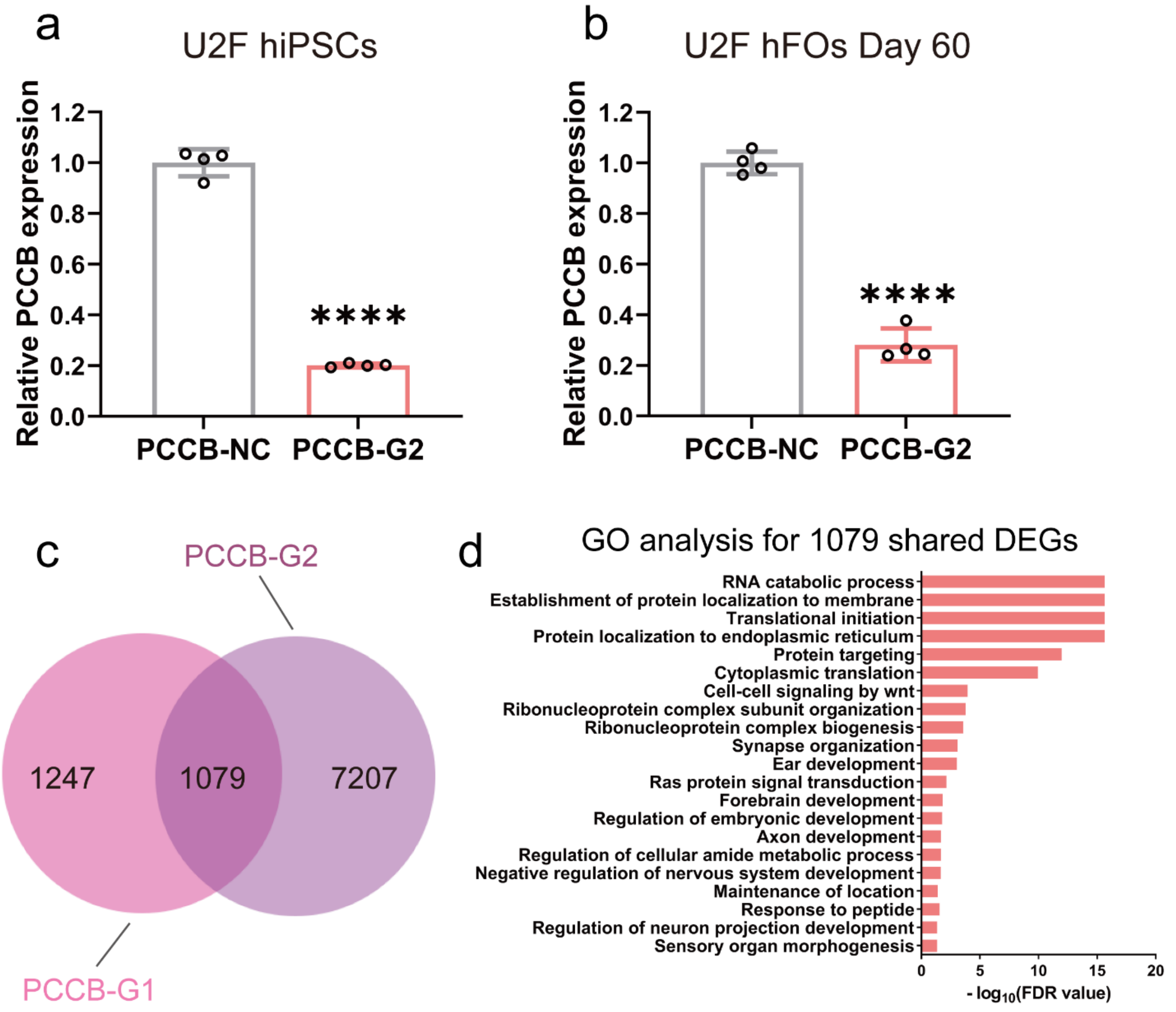

Supplementary Fig. S2. Effects of *PCCB* knockdown by *PCCB*-G2 gRNA in U2F hFOs. **a, b** RT-qPCR analysis for *PCCB* expression in U2F hiPSCs (**a**) and hFOs (**b**) after *PCCB* knockdown by *PCCB*-G2 gRNA. At least three technical replicates per group were used for the RT-qPCR analysis. Data are shown as Mean ± SD. Unpaired two-tailed t-test, ****$P < 0.0001$. **c** Overlap between DEGs identified in *PCCB*-G1 and *PCCB*-G2 hFOs. **d** GO enrichment analysis for 1079 shared DEGs between *PCCB*-G1 and *PCCB*-G2 hFOs. Source data underlying a-b are provided as a Source Data file.

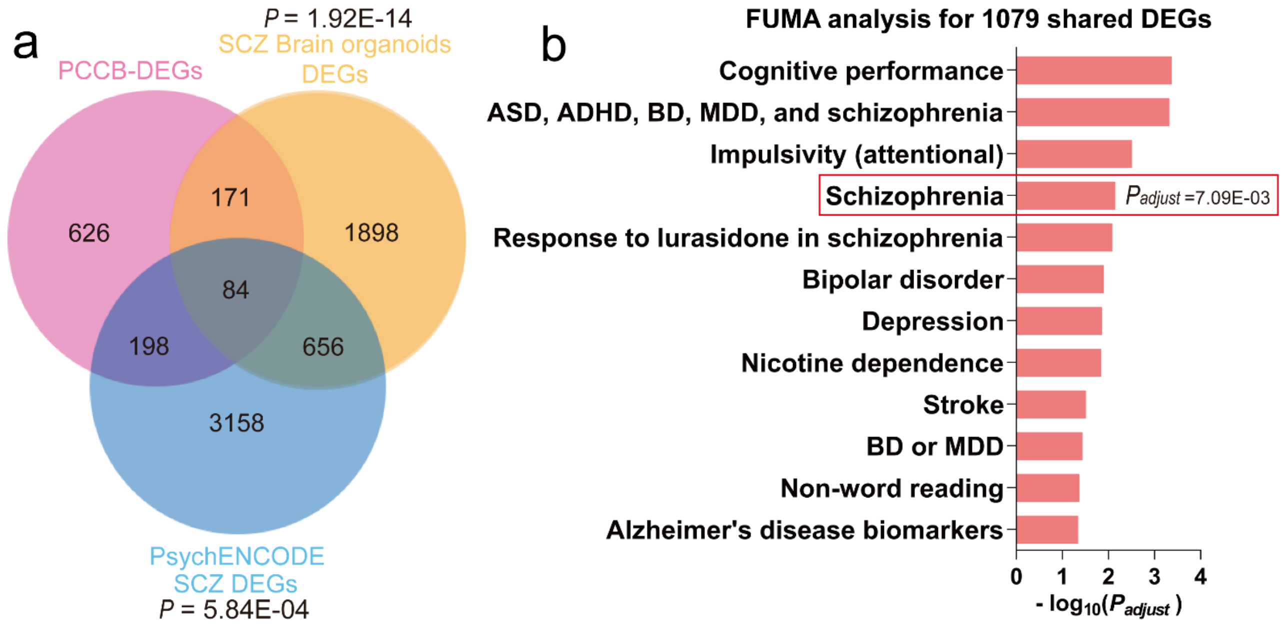

Supplementary Fig. S3. Enrichment of 1079 *PCCB* knockdown-induced DEGs with SCZ-related genes.

a 1079 *PCCB* knockdown-induced DEGs were significantly overlapped with genes dysregulated in PsychENCODE SCZ brains and SCZ patient-derived cerebral organoids. **b** 1079 *PCCB* knockdown-induced DEGs were significantly overlapped with genes reported in SCZ GWAS from the FUMA analysis. Only items related to brain diseases were displayed. *P* values are calculated by the hypergeometric test, a *P* value less than 0.05 is considered statistically significant.

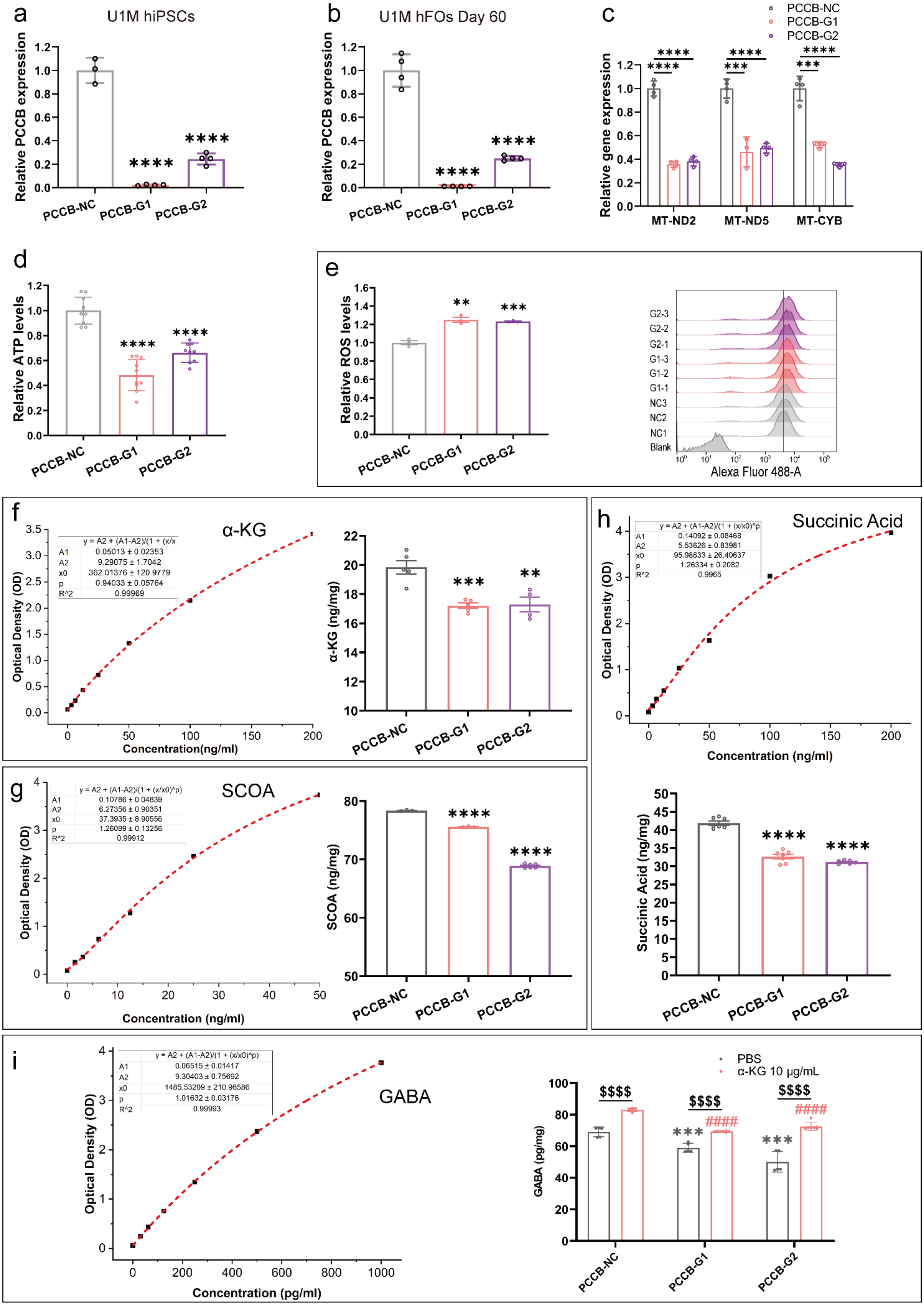
a
U1M hiPSCs
Relative PCCB expression
PCCB-NC
PCCB-G1
PCCB-G2
****
b
U1M hFOs Day 60
Relative PCCB expression
****
c
PCCB-NC
PCCB-G1
PCCB-G2
Relative gene expression
MT-ND2
MT-ND5
MT-CYB
****
***
d
Relative ATP levels
e
Relative ROS levels
**
***
G2-3
G2-2
G2-1
G1-3
G1-2
G1-1
NC3
NC2
NC1
Blank
Alexa Fluor 488-A
f
α-KG
y = A2 + (A1-A2)/(1 + (x/x
A1 0.05013 ± 0.02353
A2 9.29075 ± 1.7042
x0 362.01376 ± 120.9779
p 0.94033 ± 0.05764
R^2 0.99969
Optical Density (OD)
Concentration(ng/ml)
α-KG (ng/mg)
g
SCOA
y = A2 + (A1-A2)/(1 + (x/x0)^p)
A1 0.10786 ± 0.04839
A2 6.27356 ± 0.90351
x0 37.3935 ± 8.90556
p 1.26099 ± 0.13256
R^2 0.99912
Concentration (ng/ml)
SCOA (ng/mg)
h
Succinic Acid
y = A2 + (A1-A2)/(1 + (x/x0)^p)
A1 0.14092 ± 0.08468
A2 5.53626 ± 0.83981
x0 95.98633 ± 26.40637
p 1.26334 ± 0.2082
R^2 0.9965
Succinic Acid (ng/mg)
i
GABA
y = A2 + (A1-A2)/(1 + (x/x0)^p)
A1 0.06515 ± 0.01417
A2 9.30403 ± 0.75692
x0 1485.53209 ± 210.96586
p 1.01632 ± 0.03176
R^2 0.99993
Concentration (pg/ml)
PBS
α-KG 10 μg/mL
GABA (pg/mg)
$$$$
####

Supplementary Fig. S4. *PCCB* knockdown leads to mitochondrial dysfunction and decreased GABA levels in U1M hFOs.

a, **b** RT-qPCR analysis for *PCCB* expression in U1M hiPSCs (**a**) and hFOs (**b**) after *PCCB* knockdown by *PCCB*-G1 and *PCCB*-G2 gRNA. **c** RT-qPCR analysis for mitochondrial genes (*MT-ND2*, *MT-ND5*, and *MT-CYB*) in U1M hFOs after *PCCB* knockdown. At least three technical replicates per group were used for the RT-qPCR analysis. Data are shown as Mean ± SD. **d, e** Decreased ATP synthesis (**d**) and increased ROS content (**e**) were observed in *PCCB* knockdown hFOs. **f-h** ELISA analysis confirmed the reduction of α-KG (**f**), SOCA (**g**), and succinic acid (**h**) in *PCCB* knockdown hFOs. **i** Adding α-KG (10 μg/ml) into the culture medium of *PCCB* knockdown hFOs restored the GABA levels. At least three biological replicates were used in each group. Data are shown as Mean ± SEM. Unpaired two tailed t-test, $^{**}P < 0.01$, $^{***}P < 0.001$, $^{****}P < 0.0001$, $^{\#\#\#\#}P < 0.0001$, $^{\$\$\$\$}P < 0.0001$. Source data underlying a-i are provided as a Source Data file.

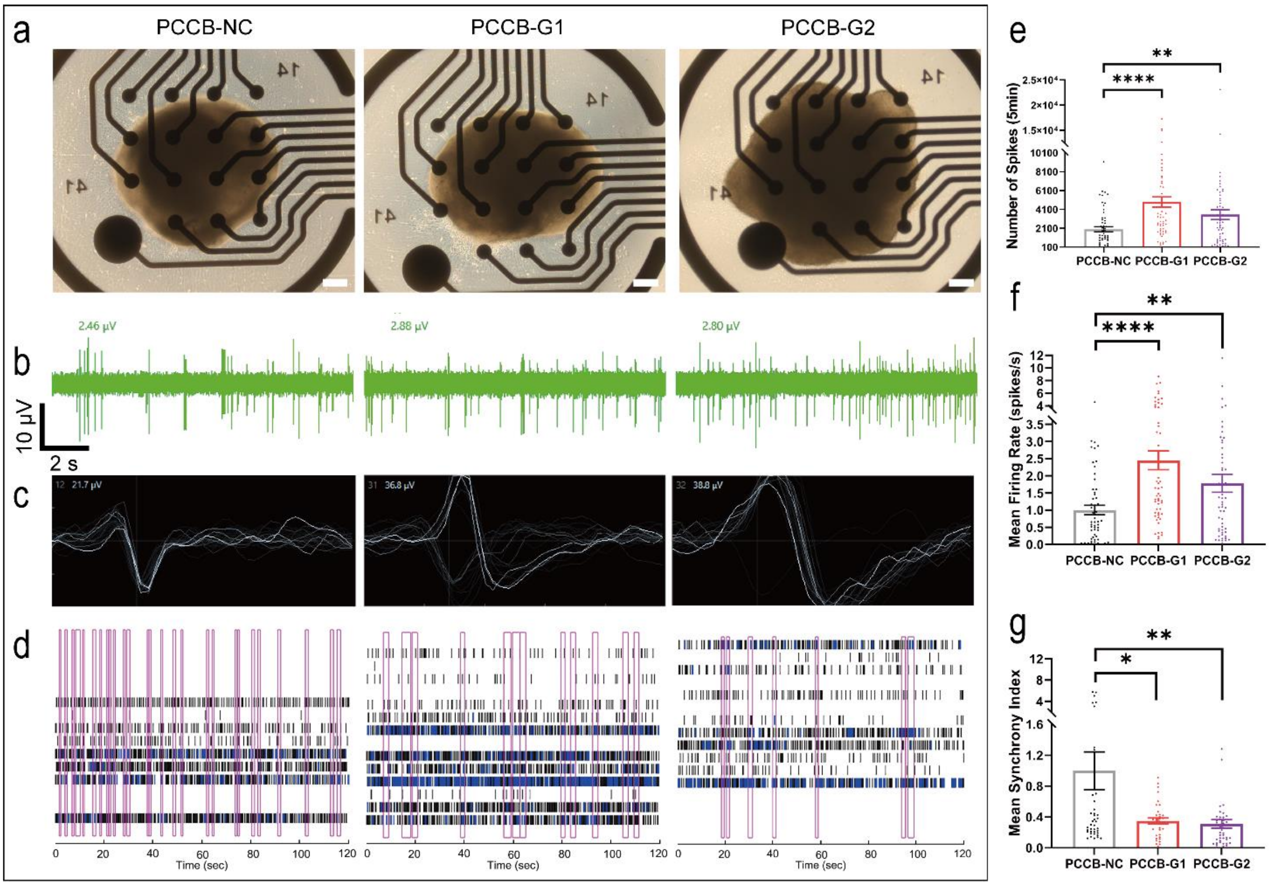

Supplementary Fig. S5. MEA assay after *PCCB* knockdown in U1M hFOs.

a Bright field of U1M hFOs cultured in 24-well MEA plate. Scale bar, 100 μm. **b** Representative burst traces for individual electrode recorded in U1M hFOs. **c** Schematic diagram of a single unit event. **d** Raster plot of synchronized burst activity. Each pink box represents a synchronized burst. **e-g** *PCCB* knockdown led to increased number of spikes (**e**) and mean neuron firing rate (**f**) but reduced synchronized burst index in U1M hFOs (**g**). Data are shown as Mean ± SEM (averaged 8 biological replicates in each group). The unpaired two-tailed t-test was used to assess difference between the *PCCB*-NC and *PCCB*-G1 or *PCCB*-G2 group. *$P < 0.05$, **$P < 0.01$, ****$P < 0.0001$. Source data underlying e-g are provided as a Source Data file.

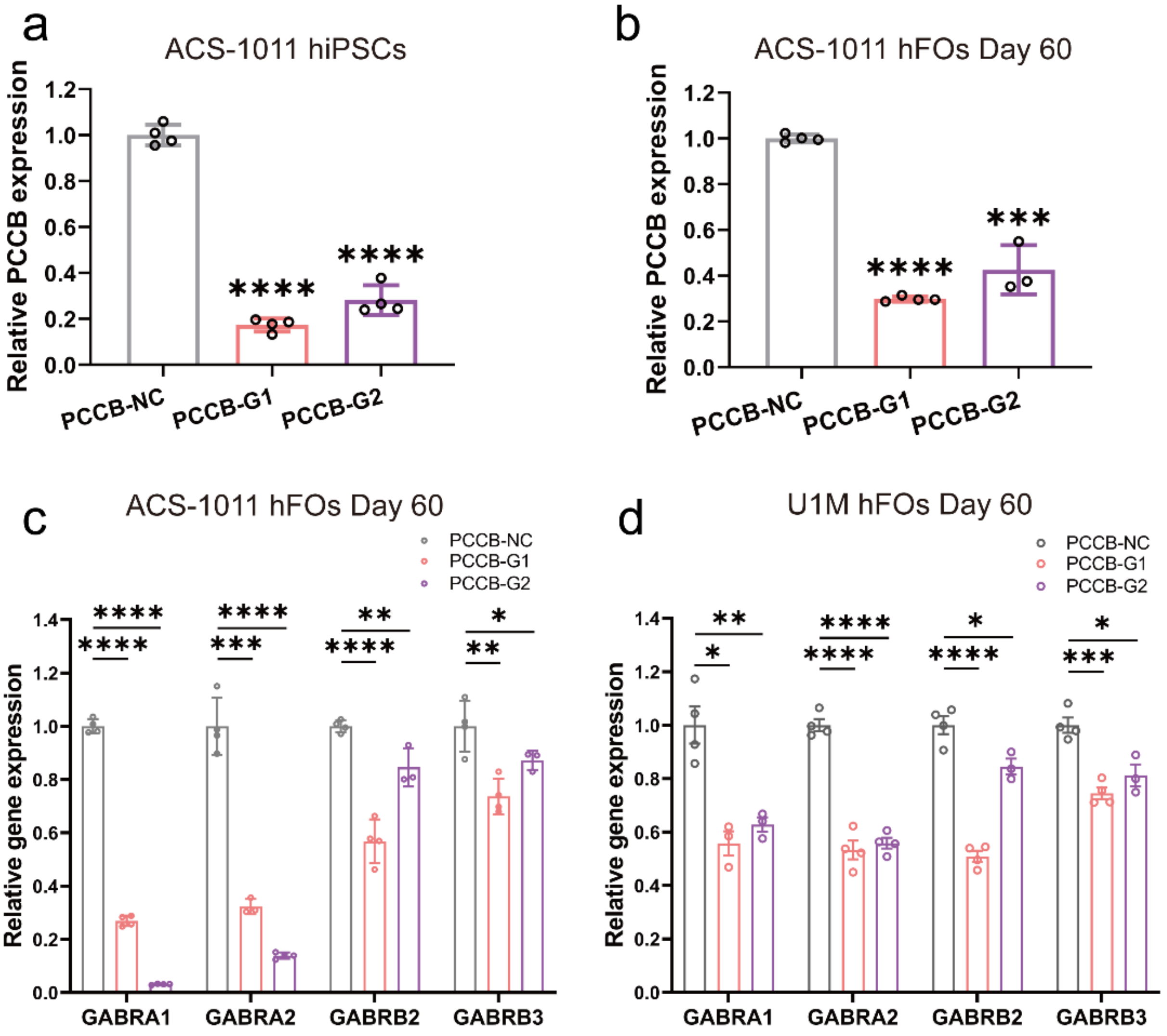

Supplementary Fig. S6. Expression of GABA receptor genes in ACS-1011 and U1M hFOs.

a, **b** RT-qPCR analysis for *PCCB* expression in ACS-1011 hiPSCs (**a**) and hFOs (**b**) after *PCCB* knockdown by *PCCB*-G1 and *PCCB*-G2 gRNA. **c, d** RT-qPCR analysis for GABA receptor genes (*GABRA1*, *GABRA2*, *GABRB2*, and *GABRB3*) in *PCCB* knockdown ACS-1011 hFOs (**c**) and U1M hFOs (**d**). RT-qPCR analysis was conducted in at least three technical replicates. Data are shown as Mean ± SD. The unpaired two-tailed t-test was used to assess difference between the *PCCB*-NC and *PCCB*-G1 or *PCCB*-G2 group. **P* < 0.05, ***P* < 0.01, ****P* < 0.001, *****P* < 0.0001. Source data underlying a-d are provided as a Source Data file.

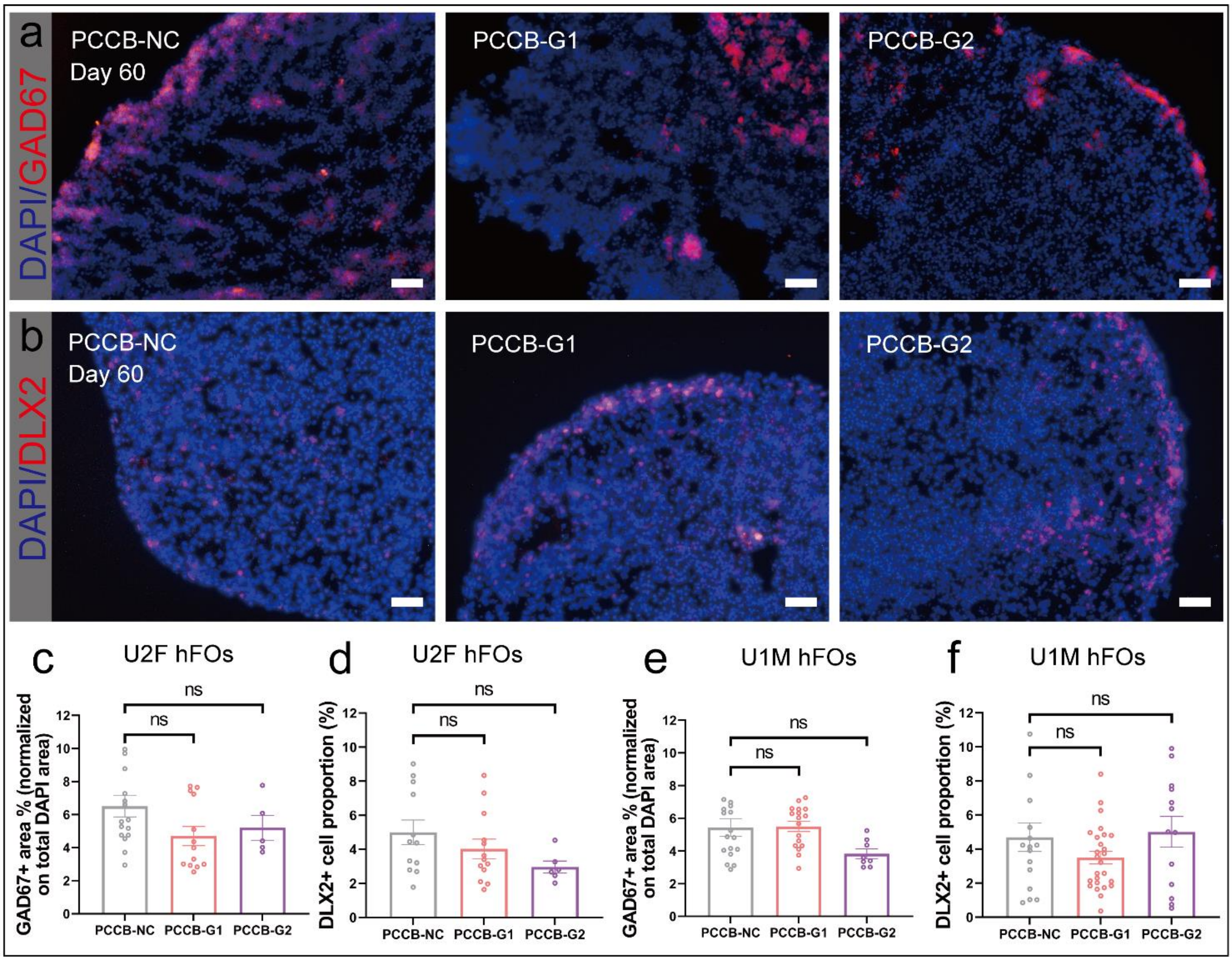

Supplementary Fig. S7. Immunostaining assay confirmed the existence of GABAergic neurons in hFOs.

a, b, Immunostaining assay was used to detect cells expressing the GABAergic neuron markers GAD67 (**a**) and DLX2 (**b**) in hFOs (day 60). Antibodies are Mouse-anti-GAD67 (Abcam, ab26116) and Mouse-anti-DLX2 (Santa Cruz, sc-393879). Scale bar, 50 μm. **c-f** Proportions of GAD67+ areas and DLX2+ cells in U2F (**c-d**) or U1M (**e-f**) hFOs were blindly quantified from 4 organoids (at least 5 organoid sections) in each group. Data are shown as Mean ± SEM. The unpaired two-tailed t-test was used to assess difference between the *PCCB*-NC and *PCCB*-G1 or *PCCB*-G2 group, ns, non-significance. Source data underlying c-f are provided as a Source Data file.

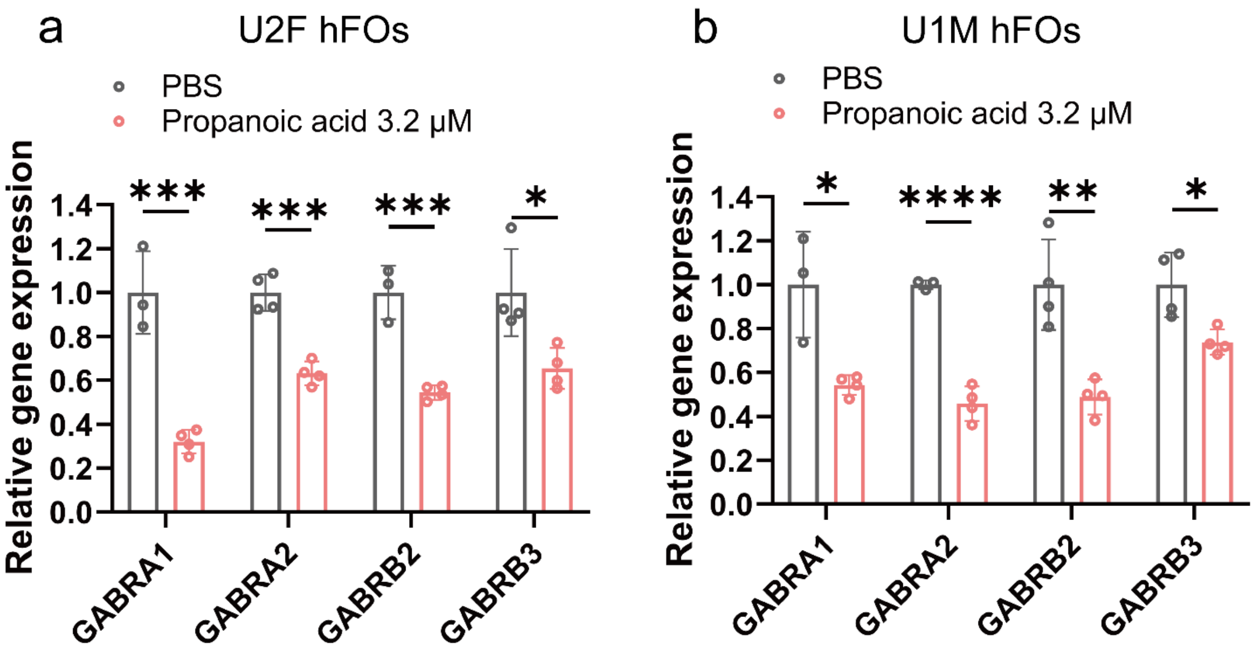

Supplementary Fig. S8. Effects of propanoic acid exposure on expression of GABA receptor genes in U1M and U2F hFOs.

a, **b** U1M and U2F hFOs were exposed to propanoic acid (3.2 μM) or PBS for 14 days. RT-qPCR was then used to detect expression of GABA receptor genes (*GABRA1*, *GABRA2*, *GABRB2*, and *GABRB3*) in U2F hFOs (**a**) and U1M hFOs (**b**). At least three technical replicates were used for the RT-qPCR analysis. Data are shown as Mean ± SD. Unpaired two tailed t-test, *$P < 0.05$, **$P < 0.01$, ***$P < 0.001$, ****$P < 0.0001$. Source data underlying a-b are provided as a Source Data file.
