# Supplementary material for: Human forebrain organoid-based multi-omics analyses of PCCB as a schizophrenia associated gene linked to GABAergic pathways
Source: Nat Commun. 2023 Aug 24;14:5176. doi: 10.1038/s41467-023-40861-2 (PMC10449845; doi:10.1038/s41467-023-40861-2)
Supplement: Supplementary file 3 — Description of Additional Supplementary Files [file 41467_2023_40861_MOESM3_ESM.pdf]

Description of Additional Supplementary Files

File Name: Supplementary Data 1
Description: Datasets used for gene prioritization

File Name: Supplementary Data 2
Description: PCCB knockdown-induced DEGs and functional analysis

File Name: Supplementary Data 3
Description: Metabolomic analysis results

File Name: Supplementary Data 4
Description: PCCB eSNP-containing DNA sequence, RT-qPCR primers and antibodies
