# Supplementary material for: Human forebrain organoid-based multi-omics analyses of PCCB as a schizophrenia associated gene linked to GABAergic pathways
Source: Nat Commun. 2023 Aug 24;14:5176. doi: 10.1038/s41467-023-40861-2 (PMC10449845; doi:10.1038/s41467-023-40861-2)
Supplement: Supplementary file 8 — Reporting Summary [file 41467_2023_40861_MOESM8_ESM.pdf]

nature portfolio

Corresponding author(s): Chunyu Liu, Chao Chen & Qingtuan Meng

Last updated by author(s): Jul 5, 2023

Reporting Summary

Nature Portfolio wishes to improve the reproducibility of the work that we publish. This form provides structure for consistency and transparency in reporting. For further information on Nature Portfolio policies, see our Editorial Policies and the Editorial Policy Checklist.

Statistics

For all statistical analyses, confirm that the following items are present in the figure legend, table legend, main text, or Methods section.

n/a	Confirmed	
☐	☒	The exact sample size (*n*) for each experimental group/condition, given as a discrete number and unit of measurement
☐	☒	A statement on whether measurements were taken from distinct samples or whether the same sample was measured repeatedly
☐	☒	The statistical test(s) used AND whether they are one- or two-sided<br>*Only common tests should be described solely by name; describe more complex techniques in the Methods section.*
☒	☐	A description of all covariates tested
☐	☒	A description of any assumptions or corrections, such as tests of normality and adjustment for multiple comparisons
☐	☒	A full description of the statistical parameters including central tendency (e.g. means) or other basic estimates (e.g. regression coefficient) AND variation (e.g. standard deviation) or associated estimates of uncertainty (e.g. confidence intervals)
☐	☒	For null hypothesis testing, the test statistic (e.g. *F*, *t*, *r*) with confidence intervals, effect sizes, degrees of freedom and *P* value noted<br>*Give P values as exact values whenever suitable.*
☒	☐	For Bayesian analysis, information on the choice of priors and Markov chain Monte Carlo settings
☒	☐	For hierarchical and complex designs, identification of the appropriate level for tests and full reporting of outcomes
☒	☐	Estimates of effect sizes (e.g. Cohen's *d*, Pearson's *r*), indicating how they were calculated

Our web collection on statistics for biologists contains articles on many of the points above.

Software and code

Policy information about availability of computer code

Data collection	Zen 2.6 (blue edition) was used to capture immunostaining figures.<br>AxIS Navigator (v3.6.2.2) was used for collecting Microelectrode array (MEA) data.<br>Gen5 (v3.11.19) was used to measure ATP content.<br>QuanStudio 3 (v1.5.2) was used to collect RT-qPCR data.
Data analysis	RNA-seq data analysis:<br>FastQC (v0.20.0)<br>STAR (v2.7.9a)<br>RSEM (v1.3.0)<br>edgeR (v3.36.0)<br>SVA (v3.42.0)<br>DEseq2 (v1.34.0)<br><br>Protein-protein interaction data analysis:<br>STRING (v11.5, online)<br>Cytoscape (v3.9.1)<br><br>Flow cytometry data analysis:<br>FlowJo (vX 10.0.7r2)<br><br>MEA data analysis:

Neural Metric Tool (v3.2.5)
AxIS Navigator (v3.6.2.2)

Metabolomic data analysis:
MetaboAnalyst 5.0 (online)

Quantification and statistics:
GraphPad Prism (v9.0.0)
Origin Pro 2018C SR1 b9.5.1.195

For manuscripts utilizing custom algorithms or software that are central to the research but not yet described in published literature, software must be made available to editors and reviewers. We strongly encourage code deposition in a community repository (e.g. GitHub). See the Nature Portfolio guidelines for submitting code & software for further information.

Data

Policy information about availability of data

All manuscripts must include a data availability statement. This statement should provide the following information, where applicable:

- Accession codes, unique identifiers, or web links for publicly available datasets
- A description of any restrictions on data availability
- For clinical datasets or third party data, please ensure that the statement adheres to our policy

RNA-seq data generated in this study have been deposited in the Gene Expression Omnibus under accession code GSE226233 (https://www.ncbi.nlm.nih.gov/geo/query/acc.cgi?acc=GSE226233). Because the raw data of the MEA and metabolomic data are huge and presented in highly diverse nature and formats, these raw data are available on request from the corresponding authors. All the other data associated with this study are shown in the manuscript, Supplementary Information, and Source Data file. Source data are provided with this paper. Public database: STRING (https://cn.string-db.org/), WebGestalt (https://www.webgestalt.org/option.php), MetaboAnalyst 5.0 (https://www.metaboanalyst.ca/).

Research involving human participants, their data, or biological material

Policy information about studies with human participants or human data. See also policy information about sex, gender (identity/presentation), and sexual orientation and race, ethnicity and racism.

Reporting on sex and gender	N/A
Reporting on race, ethnicity, or other socially relevant groupings	N/A
Population characteristics	N/A
Recruitment	N/A
Ethics oversight	N/A

Note that full information on the approval of the study protocol must also be provided in the manuscript.

Field-specific reporting

Please select the one below that is the best fit for your research. If you are not sure, read the appropriate sections before making your selection.

☒ Life sciences ☐ Behavioural & social sciences ☐ Ecological, evolutionary & environmental sciences

For a reference copy of the document with all sections, see nature.com/documents/nr-reporting-summary-flat.pdf

Life sciences study design

All studies must disclose on these points even when the disclosure is negative.

Sample size	Sample size was determined according to standard practices in this field of research and based on previous publication (PMID:28321286, PMID:34567661, PMID:35351869).
Data exclusions	hFOs were excluded from the experiments based on the following pre-established criteria:<br>The hFOs failed to grow during the 15-days expansion phase in the expansion medium.<br>The hFOs showed multiple clearly visible vacuoles.<br><br>RNA-seq data analysis:<br>RNA samples with RNA integrity number less than 7 were excluded.<br>Outlier samples were excluded based on the principal component analysis.

	MEA data analysis:<br>hFOs with number of spikes less than 5 per minute were excluded.
Replication	All experiments were not replicated but they included sufficient number of biological replicates to generate robust results.<br>The number of biological or technical replicates for all experiments is stated in the figure legends.<br>The RNA-seq, metabolomic analysis, ATP, ROS, MEA, and ELISA experiments were performed with at least three biological replicates.<br>The RT-qPCR experiments were performed with three or more technical replicates.
Randomization	In CRISPRi expriment, hiPSCs were randomly divided into the indicated groups for the lentiviral infection. For RNA-seq, RT-qPCR, metabolomic analysis, ATP, ROS, MEA, and ELISA experiments, hFOs in PCCB knockdown or control group were randomly selected for the experiments.
Blinding	For all experiments, blindness was not used in the group organization, treatment, and sample collection, but it was blinded to all groups when collecting the data and generating output.

Reporting for specific materials, systems and methods

We require information from authors about some types of materials, experimental systems and methods used in many studies. Here, indicate whether each material, system or method listed is relevant to your study. If you are not sure if a list item applies to your research, read the appropriate section before selecting a response.

Materials & experimental systems

n/a	Involved in the study	
☐	☒	Antibodies
☐	☒	Eukaryotic cell lines
☒	☐	Palaeontology and archaeology
☒	☐	Animals and other organisms
☒	☐	Clinical data
☒	☐	Dual use research of concern
☒	☐	Plants

Methods

n/a	Involved in the study	
☒	☐	ChIP-seq
☐	☒	Flow cytometry
☒	☐	MRI-based neuroimaging

Antibodies

Antibodies used	Ki67, Cell Signaling Technologies, 9449, mouse, 1:1000<br>FOXG1, Abcam, ab18259, rabbit, 1:100<br>TBR2, Cell Signaling Technologies, 81493, rabbit, 1:800<br>SOX2, Invitrogen, MA1-014, mouse, 1:200<br>PAX6, Proteintech, 12323-1-AP, rabbit, 1:100<br>MAP2, Proteintech, 17490-1-AP, rabbit, 1:200<br>GAD67, Abcam, ab26116, mouse, 1:200<br>DIX2, Santa cruz, sc-393879, mouse, 1:200<br>Alexa Fluor 594-conjugated goat anti-mouse IgG, Abcam, ab150116, 1:300<br>Alexa Fluor 594-conjugated goat anti-rabbit IgG, Invitrogen, A-11012, 1:200<br>Alexa Fluor 488-conjugated goat anti-rabbit, Invitrogen, A-11008, 1:200<br>Goat anti-mouse Cy3, BOSTER, BA1031, 1:200<br>Goat anti-mouse CoraLite488, Proteintech, SA00013-1, 1:200<br>All antibodies used in this study were also shown in Supplementary Data S4.
Validation	All primary antibodies used in this study have been validated and used for brain organoid immunostaining in our previous studies (Meng et al., Mol Psychiatry 2020; Meng et al., Transl psychiatry 2022) and other many published studies (such as Lancaster et al., Nature 2013; Quadrato et al., Nature 2017; Forsthofer et al., J Neurol 2023; Sanchez-Priego et al., Cell Reports 2022).

Eukaryotic cell lines

Policy information about cell lines and Sex and Gender in Research

Cell line source(s)	U2F and U1M hiPSCs was obtained from the Cellapy Technology (Beijing, China).<br>ACS-1011 hiPSCs was obtained from the American type culture collection (ATCC).
Authentication	All hiPSCs lines were used for karyotype analysis and were tested for the expression of pluripotency markers using immunostaining as described in our previous studies (Meng et al., Mol Psychiatry 2020; Meng et al., Transl psychiatry 2022).
Mycoplasma contamination	All cell lines used in this study have been tested for mycoplasma contamination using Mycoplasma test kit (Vazyme, D101-01). There was no mycoplasma contamination.
Commonly misidentified lines<br>(See ICLAC register)	No such cell lines were used in this study.

Flow Cytometry

Plots

Confirm that:

☒ The axis labels state the marker and fluorochrome used (e.g. CD4-FITC).

☒ The axis scales are clearly visible. Include numbers along axes only for bottom left plot of group (a 'group' is an analysis of identical markers).

☐ All plots are contour plots with outliers or pseudocolor plots.

☐ A numerical value for number of cells or percentage (with statistics) is provided.

Methodology

Sample preparation	The ROS Assay Kit (Beyotime, S0033S) was used to measure reactive oxygen species (ROS) levels of hFOs. hFOs (three hFOs were randomly pooled together as one mixed sample) at day 60 were dissociated into single cells using Accutase. After centrifuging at 1500 rpm for 5 minutes, cells were resuspended with pre-warmed serum-free DMEM-F12. The collected cells were incubated with 10 μM DCFH-DA fluorescent probes in serum-free DMEM-F12 for 20-30 min at 37°C. Cells were then analyzed on the BD FACSAria II flow cytometry. Experiments were conducted in 3-4 biological replicates. Each sample was set up with at least three technical replicates.
Instrument	BD FACSAria II flow cytometry.
Software	The data were collected by the FACSDiva software and were analyzed by the FlowJo (vX 10.0.7r2) software.
Cell population abundance	10000 cells were analyzed on the flow cytometry.
Gating strategy	Cell population in hFOs were initially identified based on the FSC/SSC. A blank control was set up to monitor the effectiveness of the fluorescent probes. The ROS level was determined by the percentage of cells labeled with fluorescence.

☒ Tick this box to confirm that a figure exemplifying the gating strategy is provided in the Supplementary Information.
